# Genomic evolution of SARS-CoV-2 in Morocco: Insights from whole genome sequences collected from 2020 to 2024

**DOI:** 10.1016/j.virusres.2025.199530

**Published:** 2025-01-31

**Authors:** Hamza GHAMMAZ, Marouane MELLOUL, Ahlam MBARKI, Mouhssine HEMLALI, Taha CHOUATI, Hicham EL ANNAZ, Nadia TOUIL, Mostafa ELOUENNASS, Khalid ENNIBI, Elmostafa EL FAHIME

**Affiliations:** a Molecular Biology and Functional Genomics Platform, National Centre for Scientific and Technical Research (CNRST), Rabat, Morocco; b Genomic Centre for Human Pathologies (GENOPATH), Neuroscience and Neurogenetics Research Team, Faculty of Medicine and Pharmacy, University Mohammed V, Rabat, Morocco; c Cell Culture Unit, Center of Virology, Infectious and Tropical Diseases, the Mohammed V Military Training Hospital, Rabat, Morocco; d Immunopathology Research Team (ERIP), Faculty of Medicine of Pharmacy, University Mohammed V, Rabat, Morocco; e Department of Bacteriology, Mohammed V Military Teaching Hospital, Rabat, Morocco; f Microbiology and Molecular Biology Team, Center of Plant and Microbial Biotechnology, Biodiversity, and Environment, Faculty of Sciences, Mohammed V University of Rabat, Rabat, Morocco; g University Mohammed VI of Science and Health (UM6SS), Casablanca, Morocco; h Genomics and Molecular Biology, Mohammed Vi Center for Research and Innovation (CM6RI), Rabat, Morocco

**Keywords:** SARS-CoV-2 variants, clades, Morocco, Genomic surveillance in Morocco

## Abstract

This study investigates the evolution and genetic diversity of SARS-CoV-2 strains circulating in Morocco to track the spread, clade distributions and mutations of the virus across various regions from February 2020 to June 2024. The genome sequences were retrieved from the GISAID database. A total of 2630 SARS-CoV-2 genome sequences were analyzed using bioinformatic tools such as Nextclade, followed by phylogenetic and statistical analyses. The study highlights the predominance of the GRA clade (Omicron variant) since November 2021, while clades such as G, GH, GR, and GRY were identified earlier. The GRA clade exhibited the highest number of non-synonymous mutations, particularly in the Spike (S) gene, suggesting strong evolutionary pressure. The correlation analysis between structural and non-structural proteins revealed key interactions between S and NSP5, providing insights into the viral replication and assembly processes. This work gives new insights to the dynamics of SARS-CoV-2 in Morocco and underscores the importance of ongoing genomic surveillance to respond to emerging variants and potential future outbreaks.

## Highlights

• A total of 1989 whole genome sequences of SARS-CoV-2 viruses circulating in morocco from 2020 to 2024 were analyzed.
• The omicron variant (GRA clade) became the dominant strain in morocco since november 2021, exhibiting the highest number of non-synonymous mutations, mainly in the structural protein s (Spike).
• Phylogenetic analyses revealed the emergence and transmission patterns of SARS-CoV-2 variants within Morocco, with ties to other regions such as Europe, mainly england.
• A positive correlation (74%) between mutations occurring in s and NSP5 genes offered new insights into viral mechanism evolution.

## 1 Introduction

In late December 2019, a novel coronavirus SARS-CoV-2 has emerged in China and has spread to multiple countries worldwide (Gorbalenya et al., 2020) leading to coronavirus disease 2019 (Covid-19). The virus has been spread globally and infected around 775 million people cases including more than 7 million deaths by the end of June 2024.

The African continent has been relatively spared by the COVID-19 pandemic; there were 12 553 199 cumulative cases and a total of 259 265 deaths (CDC, 2024). In 2 March 2020, the first report of SARS-CoV-2 virus infection was declared in Morocco, and as of the date of publication, there have been 1.26 million Covid-19 cases, with 16,237 deaths (Moroccan Ministry of Health, 2024).

The Moroccan Ministry of Health created the National Scientific Committee and developed a health strategic action plan soon after the declaration of COVID-19 as pandemic (MATIN, 2021). Throughout the following months, the committee established a national network to strengthen genomic surveillance efforts at country level and to rapidly respond to potential outbreaks of SARS-CoV-2 in all 12 regions of the country. Efforts included sample collection, diagnostics, data sharing and analysis.

Covid-19 infection was first declared on March 02, 2020 in Morocco introduced by a Moroccan expatriate residing in Bergamo province, Italy (Crisis24, 2020). A second case was confirmed later that same day involving an 89-year-old Moroccan woman residing in Italy who had returned to Morocco on 25 February from Bologna, Italy (Kasraoui, 2020). Ten days later, five cases were declared positive coming from European countries (Spain, Italy and France) to different locations (Casablanca, Marrakech and Tangier). Patients presented common signs of Covid-19 including breathing difficulties, cough, cephalalgia and stomach-ache. All patient's laboratory-confirmed wild-type SARS-CoV-2 infection at the national public health institute (INH) (CDC) and further confirmed by the Pasteur Institute of Casablanca.

Following this, the country has taken a series of measures to prevent the spread of the virus. Schools and universities closed on 13 March 2020 while air and sea connections with Europe were suspended three days after the first case was confirmed.

Morocco's leading genomic researchers were urgently advocating for a coordinated approach to genomic surveillance of SARS-CoV-2 in the country's most vulnerable settings, especially the Eastern and Southern parts of the kingdom (Oujda, Ouarzazate, Guelmim, Laayoune and Dakhla).

The National Center for Scientific Research (CNRST) together with the INH, Laboratoire de Recherche & d'Analyses Medicales de la Gendarmerie Royale, the centre of Virologie & Maladies Infectieuses Tropicales of Rabat and Pasteur Institute of Casablanca form the SARS-CoV-2 Genomics Consortium in April 2020. This consortium aims at monitoring SARS-CoV-2 evolution as part of genetic characterization and identifies the predominant genetic sequence among the many SARS-CoV-2 viruses.

In the present study, we joined analyses of SARS-CoV-2 genomes sequences to get an updated picture of the local circulating strains, to track SARS-COV-2 epidemics in Morocco and monitor cross border transmission over a 4-year period (2020–2024).

We have investigated the Global Initiative for Sharing All Influenza Data (GISAID) database (Shu and McCauley, 2017) and a special focus on the Omicron variant, its lineages and hybrid variants are presented.

We successfully traced the evolutionary alterations of the SARS-CoV-2 subsequent to its initial introduction in the country.

## 2 Materials and methods

### 2.1 Data source and selection of sequences

SARS-CoV-2 sequences were retrieved by accessing GISAID website using a multitude of sorting and personalized queries. Under search menu of the EpiCov genome browser, Africa/Morocco was selected in the location filter with date of collection in year-month-day format for the period between 2nd February 2020 and 30th June 2024.

A total of 2630 genomic sequences were retrieved from GISAID in Fasta format.

Later on, we have used Nextclade quality check to remove bad quality or low coverage sequences. A total of 2026 sequences with complete genomes were selected of which, 37 sequences were removed because of bad deletions. We selected a final filtered fasta file containing 1989 sequences (Flowchart 1).

**Flowchart 1:**
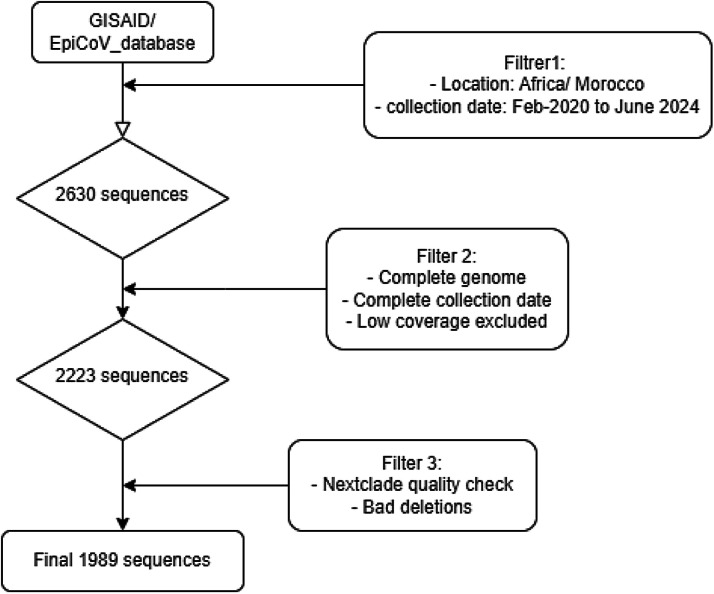
Data source and workflow analyses of sequences.

### 2.2 Bioinformatic analysis

Sequences were aligned to the reference genome (Wuhan-Hu-1/2019 (MN908947)) using Nextclade service in the Nextstrain website (Aksamentov et al., 2021).

We have used three python scripts (aa_frame_0.py, aa_frame_1.py and aa_frame_2.py) and all mutations were extracted from the aligned Fasta file, including the nucleic acid change, the amino acid change with their positions on the genome and in the protein respectively.

Three files are then generated (aa_frame_0.csv, aa_frame_1.csv and aa_frame_2.csv), on which AWK programming language was used to extract genes with the correct reading frame from each file (Table 1).

**Table 1:** summary table of SARS_CoV_2 genes with their correct reading frame.

Frame_0	Frame_1	Frame_2
nsp12, nsp13, nsp14, nsp15, nsp16, ns3a, E, ns6, ns7a	nsp1, nsp2, nsp3, nsp4, nsp5, nsp6, nsp7, nsp8, nsp9, nsp10, nsp11, S, N, ns10	M, ns7b, ns8

Afterwards, each folder was split into two files, one contains synonymous mutation (S) and non-synonymous (NS) resulting in 6 files (F0S.csv, F1S.csv, F2S.csv, F0NS.csv, F1NS.csv and F2NS.csv). The S mutation files were assembled into Final_S.csv and NS ones into final_NS.csv (Flowchart 2). Deletions were extracted using Del.py script and insertions were analyzed using nextstrain website. All the scripts can be found at https://github.com/HGHAMMAZ687/Bioscript_687.git.

**Flowchart 2:**
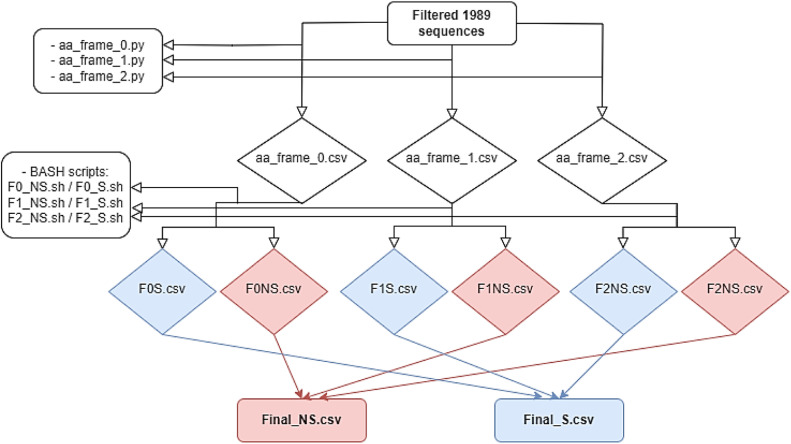
data genomic mutation extraction process Non_synonymous(NS) and Synonymous (S).

### 2.3 Phylogenetic analysis

We downloaded 2021–2024 season circulating Omicron SAS-CoV-2 strain datasets. The Phylogenetic tree displaying Omicron variant information is visualized by the Nextclade/Nextstrain (Aksamentov et al., 2021). Phylogenetic trees were constructed based on the best quality score (qc.ovellscore) of Omicron repres entative sequences for each lineage (*n* = 107). The MEGA11, neighbor joining method was used, trees are then plotted using ggtreeextra R package (ver 1.14.0). closeness to strains on the global scale was assessed using USHER (Ultrafast Sample placement on Existing tRee) https://genome.ucsc.edu/cgi-bin/hgPhyloPlace.

### 2.4 Statistical analysis

Pearson correlation test was performed on the filtered 1898 sequences for all the 27 SARS_CoV_2 genes (Nonstructural proteins (nsp1, nsp2, nsp3, nsp4, nsp5, nsp6, nsp7, nsp8, nsp9, nsp10, nsp11, nsp12, nsp13, nsp14, nsp15, nsp16, ns3a, ns6, ns7a, ns7b, ns8 and ns10), Spike(S), Nucleocapsid (N), Envelope (E), Membrane (M)) using Corrplort R package (ver 0.92). The significance of p value was set at 0.05.

## 3 Results

### 3.1 Distribution of SARS-CoV-2 identified through the genome sequence analysis from february 2020 to june 2024

On average, 76% of total genome sequences contained high to moderate -quality data according to data quality check.

**Fig. 1:**
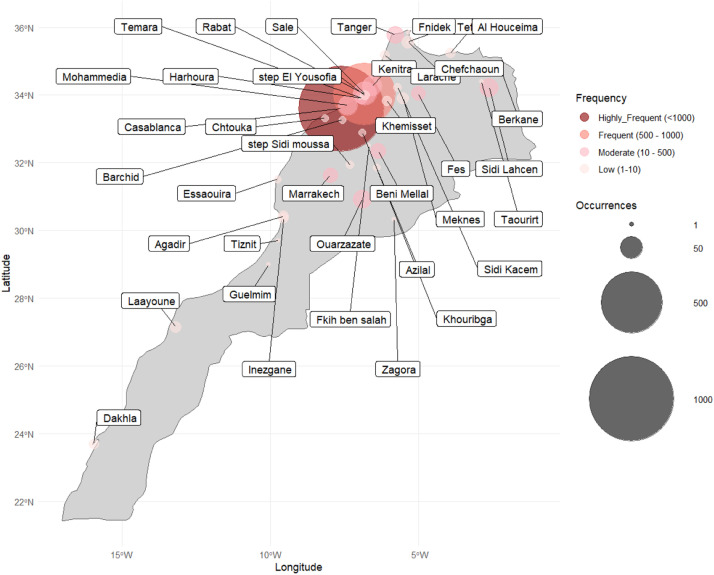
A location of human SARS-CoV-2 genome sequences redacted from GISAID database between Feb-2020 and June-2024. The map indicates the number of sequences at each location. The size of circles indicate the number of published sequences at each region.

Fig. 1 depicts the number of whole human SARS-CoV2 genomes obtained in GISAID EpiCoV database using high-throughput sequencing over the last four years (February-2020 to June-2024). A total of 1989 sequences were kept for our analyses.

**Fig. 2:**
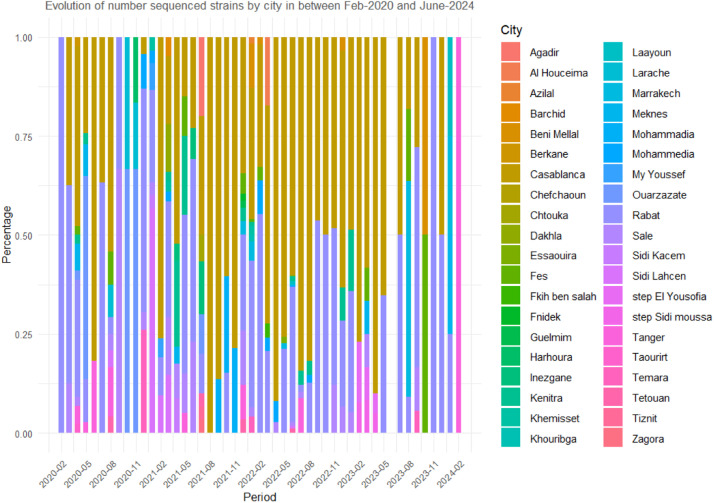
**Stacked bar plot showing the evolution of sequenced SARS-CoV-2 strains across Moroccan cities from February 2020 to June 2024.** The x-axis represents quarterly time periods, while the y-axis shows the percentage of strains sequenced by city, ranging from 0 to 100%. Each bar is divided into colored segments, with each color corresponding to a specific city, reflecting the proportion of strains sequenced from that city during the given period. The legend lists the cities and their respective colors, including Agadir, Casablanca, Marrakech, Rabat, and others, with fluctuations in representation over time.

Genome sequences were published from different locations (Fig. 2), with frequencies varying from region to region. The higher number of published genomes was reported in Casablanca-Settat and Rabat-Sale-Kenitra with 1064 and 638 sequences respectively during February-August 2020 and from February 2021 to May 2023. During September - December 2020 to mild January 2021, genome sequences were majoritarily from Marrakech-Safi, Fes-Meknes and Tanger-Tetouan-Al Hoceïma regions. No data were reported during May 2023 to June 2023.

Other regions such, Drâa-Tafilalet, Béni Mellal-Khénifra and Oujda Angad, an average of 30 genomes sequences was recorded, while considerably low sequencing data were obtained from southern regions including, Souss-Massa, Laâyoune-Sakia El Hamra, Dakhla-Oued Ed-Dahab and Guelmim-Oued Noun where an average of 7 genome sequences determined (Fig. 1).

**Fig. 3:**
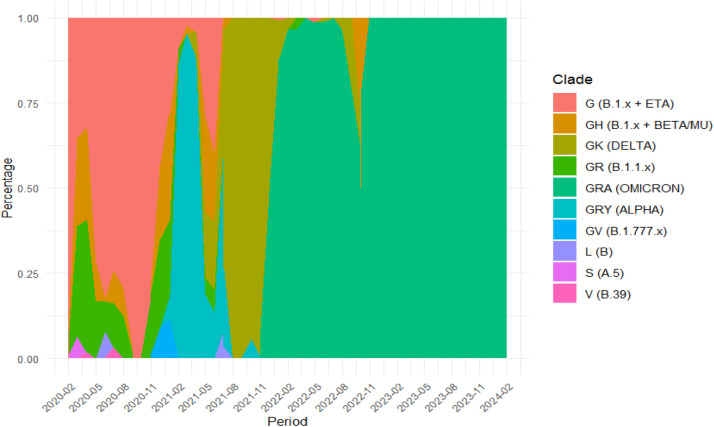
Distribution of various SARS-CoV-2 clades in Morocco February 2020 - 2024. Y-Axis: Represents the percentage, ranging from 0 to 1 (or 0% to 100%). X-Axis: Represents the time period, with intervals from February 2020 to February 2024.Different clades are represented by different colors. The clades include: G (orange),GH (red), GK (green), GR (yellow), GRA (cyan), GRY (teal), GV (blue), L (purple), S (pink), V (light pink).

The first reported sequence in GISAID from Morocco was under GISAID accession number EPI_ISL_451,400 collected on 23 April 2020 and submitted one month later. However, when we analyze Fig. 2, Fig. 3, the first genome sequences were collected on February 2020. These isolates were collected on the same day with identifiers: _ISL_4,899,898, EPI_ISL_4,899,903, EPI_ISL_4,899,863, EPI_ISL_4,899,870, EPI_ISL_4,899,911, EPI_ISL_4,899,917, EPI_ISL_4,899,892, EPI_ISL_4,899,881 and EPI_ISL_4,899,888 and released in GISAID on 10 June 2021 from INH, Rabat**.**

### 3.2 Genetic diversity, synonymous and non- synonymous mutations among SARS-CoV2 clades

When using the R program, the sequenced SARS-CoV-2 genomes were clustered into L, S, V, G, GH, GR and GV clades. The chronological distribution of SARS-CoV-2 clades is presented in Fig. 3**.** The analysis showed that clade G predominated at the beginning of the pandemic (Feb-Sept 2020), while a smaller proportion of coexisting GK and GR clades was determined. Thereafter, there was a noticeable rise in clade diversity, with the GR clade becoming more prominent and new clades evolved including the GRA and GV. Genomes that belong to GH clade experienced significant fluctuations from late 2020 to early 2021, while those in GRA clade emerged.

**Fig. 4:**
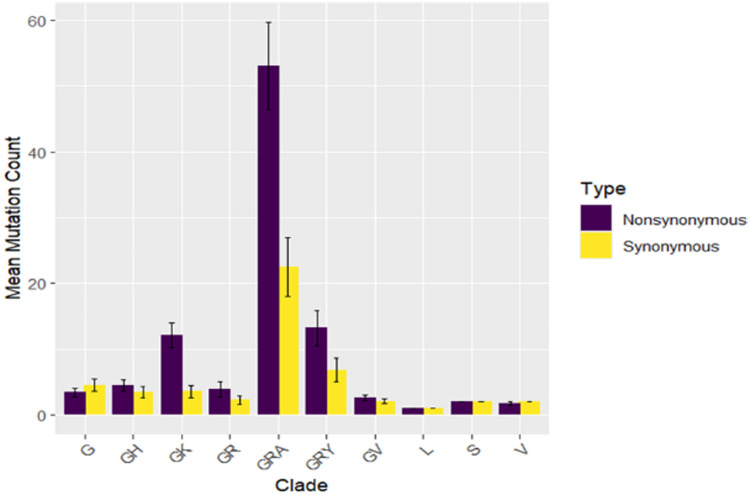
Mean ± SD of mutation counts for both synonymous and non-synonymous mutations across various SARS-CoV-2 clades.

During 2021, the GRY clade makes a substantial rise, peaking early in the year, as the GR clade's dominance wanes in favor of the GRY clade. In 2022 - 2023, the GRA clade became the dominant strain and maintained its leading position, while other clades like GH, GR, and GV declined. Currently, the GRA clade or Omicron variant continues to be the predominant strain (that includes: BA.5 (665), BA.1 (217), BA.2 (203), BQ.1 (153), XBB.1.9 (41), BA.2.12.1 (24),XBB.1.5 (20), BA.4 (18), XBB (9), XBB.2.3 (8), XBB.1.16 (5), JN.1 (3), BA.2.75 (2), CH.1.1 (1), EG.5.1 (1), XBB.1.5.70 (1), XBV (1) and XW (1)). The different plots in Fig. 4 illustrate the mean number of synonymous and non-synonymous mutations across various SARS-CoV-2 clades.

The GRA clade stands out with the highest mean mutation counts in both categories, especially non-synonymous mutations, which average close to 60 per genome, significantly higher than its synonymous mutations. This pattern indicates a strong evolutionary drive within the GRA clade, possibly due to selective pressures that favor mutations altering protein function.

The GRY clade also shows a substantial mean mutation count, with a notable but smaller gap between synonymous and non-synonymous mutations.

Other clades like GH, GK, and G have moderate mutation counts, with relatively balanced means between the two mutation types. Clades such as L, S, and V exhibit the lowest mutation counts, with very similar means for both synonymous and non-synonymous mutations.

The synonymous mutations heat map highlights the GRA clade as having the most significant number of mutations, particularly in the S gene with 3280 mutations.

**Fig. 5:**
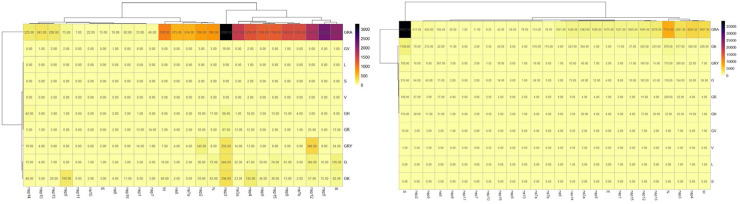
Distribution of mutations across different genes and lineages of SARS-CoV-2. (A) represents Distribution of thesynonymous mutations and (B) represents Distribution of Non-synonymous mutations. Rows corresponds to the specific gene of the SARS-CoV-2 virus and column represents a specific lineage of SARS-CoV-2. These lineages are grouped hierarchically, as shown by the dendrogram at the top of the heatmap.

Other genes like nsp12 and nsp6 also display high mutation counts across various clades, though these are generally lower than the non-synonymous mutations. The nsp16, ns7a, and E genes show relatively fewer synonymous mutations, possibly indicating higher conservation or functional constraints (Fig. 5a).

On the other hand, the non-synonymous mutations reveal that the GRA clade also exhibits the highest mutation counts across several SARS-CoV-2 genes, with an exceptionally high concentration in the S gene, where the number of mutations reaches 38,667. Other significant mutations are observed in the nsp2, nsp3, N, and ns3a genes, particularly within the GRA, GRY, and G clades (Fig. 5b).

### 3.3 Correlation test between the different SARS-CoV-2 non-structural proteins (NSPs), structural proteins, and other viral proteins

**Fig. 6:**
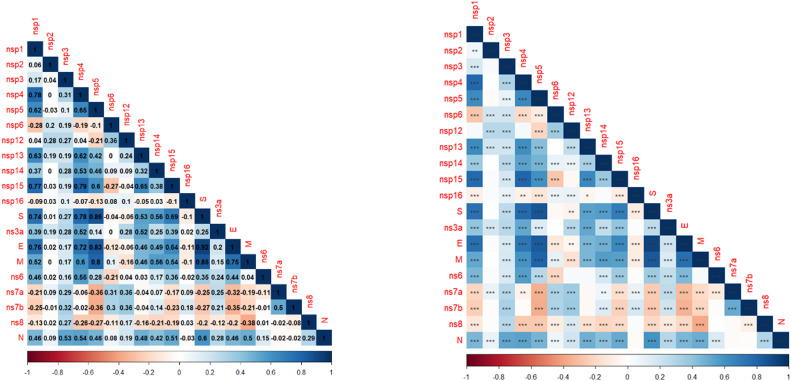
Pearson correlation test of different SARS-CoV-2 non-structural proteins (NSPs), structural proteins, and other viral proteins. (A) Correlation matrix heat map showing correlation coefficient. Each cell represents the Pearson correlation coefficient between the protein on the x-axis and the protein on the y-axis. The values range from −1 to 1, with - 1 indicating a perfect positive correlation, −1 indicating a perfect negative correlation and 0 indicating no correlation.(B) heat_map with the corresponding P-value (***:highly significant, **: very significant, *: significant).

Fig. 6 shows the correlation between different SARS-CoV-2 non-structural proteins (NSPs), structural proteins, and other viral proteins.

There are many strong positive correlations (dark blue) among NSPs. For instance, nsp1 and nsp2 (correlation of 0.62), nsp3 and nsp4 (correlation of 0.78). The structural protein S shows a strong positive correlation with several NSPs, such as nsp5 (correlation of 0.78) and nsp8 (correlation of 0.86). Negative correlations are less common but still present. For example, nsp4 and ns6 (correlation of −0.19) and nsp14 and ns7a (correlation of −0.21).

Several proteins show very low or no correlation (values near 0), indicated by white or very light-colored cells. For example, nsp12 and ns7b (correlation of 0.00) and ns6 and ns8 (correlation of −0.01).

### 3.4 Phylogenetic analysis of moroccan omicron strains

**Fig. 7:**
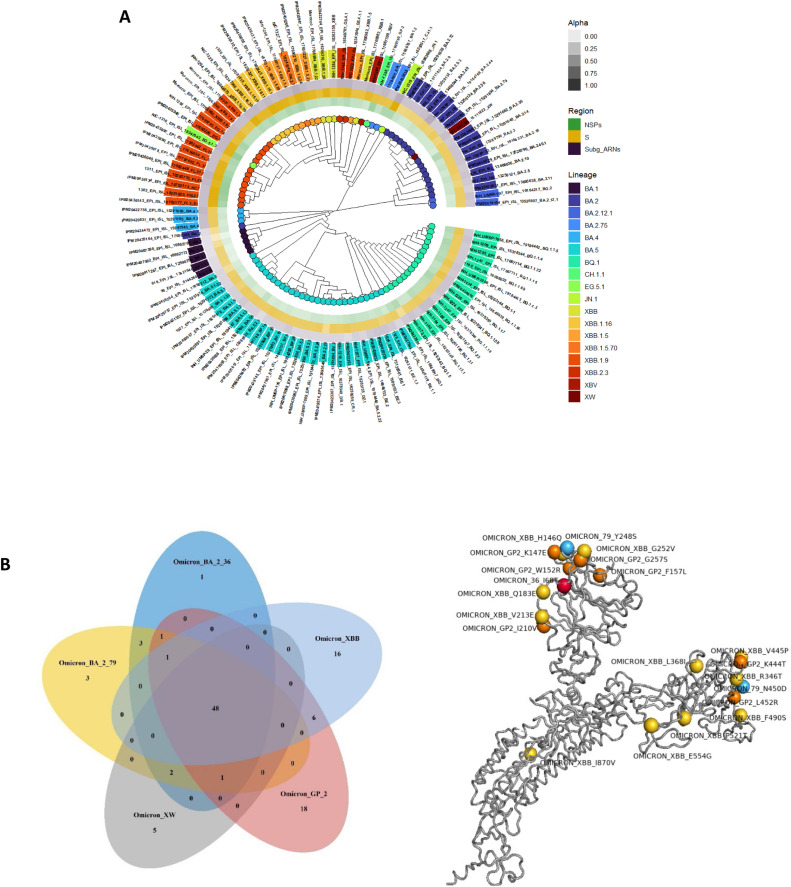
Phylogenetic analysis of Moroccan Omicron strains. (A) bar chart shows the mutation counts for various genes. Here is a detailed description and analysis: Y-Axis: Represents the mutation count, ranging from 0 to 40,000. X-Axis: Lists various genes including nsp1, nsp2, nsp3, to ns10. (B) Venn diagram showing the overlap of different Omicron variants of the SARS-CoV-2 virus: Omicron_BA_2_36, Omicron_BA_2_79, Omicron_XBB, Omicron_XW, and Omicron_GP_2 (C) Spike protein with positions of unique mutations relative to each omicron variant.

The phylogenetic tree illustrates the relationships among various Omicron variants of SARS-CoV-2 (Fig. 7). It indicates the substantial diversity within the Omicron variants, reflecting multiple sub-lineages that have evolved.

The distribution of mutation percentages across different regions of the Omicron SARS-CoV-2 genome reveals distinct patterns among the analyzed strains. On average, mutations are most prevalent in the Spike (S) protein region, with a mean of approximately 52.57%, followed by the non-structural proteins (NSPs) at 25.47%, and subgenomic RNAs (Subg_ARNs) at 21.96%.

Among the analyzed SARS-CoV-2 strains, six have been identified as significantly deviating from the majority in their mutation patterns across the NSPs, S protein, and Subgenomic RNAs (Subg_ARNs) regions. Notably, `98_EPI_ISL_8,144,260_BA.1.18` and `IPM20411287_EPI_ISL_12,590,756_BA.1.1` exhibit elevated mutation percentages in the Spike protein region, reaching nearly 60%.

The strain `IPM20407398_EPI_ISL_10,862,769_BA.1`, with a more balanced mutation pattern, is closely related to a strain from Kenya.

Meanwhile, `NIH-1244_EPI_ISL_17,667,709_XBV`, showing higher mutation rates in NSPs and a lower percentage in the Spike protein, has a nearest neighbor from England, similarly NIH-1247_EPI_ISL_17,667,711_BQ.1.1.15, another notable strain, shows a lower mutation percentage in the Spike protein at 38.78% and is closely related to a neighbor strain from England.

The XBV lineage is positioned in the phylogenetic tree between two distinct groups. On one side, it is flanked by GP.2, BN.1.2, and CJ.1.1 lineages; while on the other side, it is closely positioned next to XBB.1 and XBB.1.5.

## 4 Discussion

The present study seeks to investigate the evolution of SARS-CoV-2 genomes with respect to their geographical distribution since February 2020 until June 2024 in Morocco. Genetic diversity and amino acid mutation analysis are also determined.

Collection of SARS-CoV-2 genome sequencing data is based on bioinformatics tools publically available, while data interpretations and analyses were realized by using algorithms to select only genome sequences with high quality and extract relevant data. We assume that our data are accurate and may represent the real CoVid-19 disease evolution in Morocco.

When exploring GISAID database, the first case was declared on 02 March 2020 with accession number of EPI_ISL 476,559. The virus was sequenced from a patient returning from Bergamo, Italy, considered as the Covid-19 epicenter in Europe earlier in Covid-19 pandemic. where the largest foreign community is the one from Morocco with 14.5% (17,617) of all foreigners present on the territory (ISTAT, 2024). This corroborates the probable introduction of Covid-19 into Morocco from Italy.

Variations in frequencies of analyzed genome sequences have been noted among different locations in Morocco. The high frequencies were determined in regions with high degree of exposure to SARS-CoV-2 and with high mortality rates. These areas are the most densely populated provinces marked by a massive influx of passengers. Casablanca-Settat and Rabat-Sale-Kenitra became known to the country as the pandemic's epicenter in March 2020. In mild 2020, Fes-Meknes highlighted the highest number of deaths, while higher mortality rates were reported in Tanger-Tetouan-Al Hoceïma at the end of the year.

When the emerging SARS-CoV-2 variants were classified into clades, the distribution of the SARS-CoV-2 variants circulating in Morocco was approximately similar to that of global distributions.

The clade G was introduced in Morocco probably from Northern part of Italy (Lombardy) in February 2020. This clade contains the D614G mutation in the S spike proteins which cause a higher Infectiosity (Hoehl and Ciesek, 2020). Other clades such as GH (B.1.x) and GR (B.1.1.x) were ephemeral and as a consequence Morocco imposed a total lockdown from March to June 2020 soon after the surge of SARS-COV-2 Clades such as L, S, and V (H24info, 2020). In addition, our results demonstrated that these clades have moderate mutation counts, with relatively balanced means between synonymous and non-synonymous mutation types. This is not the same case as for clades L, S, and V; which exhibited the lowest mutation counts, with very similar means for both mutation types, suggesting the later clades as more conserved.

The clade G was the dominant clade when Morocco has lifted the lockdown by the end of 21 December 2020. Later, the clade GRY or Alpha variant (B.1.1.7) was introduced by an imported case from Ireland in January 2021 (REUTERS, 2021). At the same time, Morocco launched the first coronavirus vaccination campaign with the Oxford–AstraZeneca and Sinopharm BIBP vaccines (Africanews, 2024).

Alpha variant demonstrated higher infectivity because of mutations such N501Y, K417N/T and E484K mutants (Khan et al., 2021). At this time, this variant was already becoming the predominant clade with a global distribution of 29% and responsible for the second wave in Morocco (CNBC, 2024).

In May 2021, the clade GK or Delta variant (B.1.617.2) was notified with a global distribution of 14.5%, while in Morocco, the first two cases were declared; one involving a traveler from India and the other through local transmission. Meanwhile, 14 million vaccine doses had been administered with 35% of the target population had been fully vaccinated (Moroccan Ministry of Health, 2024). This clade recorded the highest case numbers on August 2021 accounting for 80% of infections, in parallel with a global distribution of 96% (CNBC, 2024). This variant posed concerns due to its rapid global spread and heightened transmissibility with specific mutations in addition to T478K, P681R and L452R (Tian et al., 2021).

The GRA clade Omicron has been the prevalent strain of SARS-CoV-2 virus in circulation since November 2021. In December 2021, Omicron (B.1.1.529) started to circulate in our country and Covid-19 peak began to increase. This has led to the highest proportion of infections (i.e. third wave) occurring during early January 2022. The first Omicron variant case was not considered as an imported case but as a local mutation occurring in Casablanca-Settat region. This discovery underscored the virus's ability to evolve within the country, increasing the need for ongoing vigilance and adaptation of public health strategies (Badri, 2021).

When analyzing variable nonsynonymous/synonymous mutations among the different clades, higher mutation rates were determined mainly in S, nsp2, nsp3, N, and ns3a genes. This suggests intense evolutionary pressure on these regions, possibly due to the virus's need to adapt to host immune responses or environmental factors, virulence, and ability to evade immune defenses (Carabelli et al., 2023). Interestingly, theses mutations are also described within other clades (i.e., GRY, and G). Furthermore, the strong correlations involving the S (spike protein) with NSP5 (non-structural protein 5), E (envelope protein), and M (membrane protein) highlight potential functional interactions that may be critical for viral assembly or other processes. For instance, the high correlation between S and M (0.83) and S and E (0.76) is consistent with these proteins being co-involved in viral structure formation. Also, E and M (0.92) is expected, as both proteins are involved in forming the viral envelope, likely working together closely during viral assembly (de Haan and Rottier, 2005). On the other hand, the biological correlation between NSP5 and the spike protein is intriguing, especially given their respective roles in the viral life cycle. The strong positive correlation (0.74) between NSP5 and the spike protein suggests that their expression or activity might be co-regulated during the viral life cycle. While NSP5 is primarily involved in the processing of viral polyproteins (Jin et al., 2020), and spike protein is involved in viral entry (Wrapp et al., 2020), the strong correlation hints at a possible coordinated mechanism during viral assembly and maturation. The virus likely needs to maintain a certain level of both NSP5 and spike protein for efficient viral replication and spread. During viral replication, NSP5 processes the polyprotein to yield various non-structural proteins essential for viral RNA synthesis and replication, which might, in turn, regulate structural protein production, including spike (V'kovski et al., 2021). The three asterisks (***) in the second image indicate that this correlation is highly statistically significant, suggesting that the relationship between NSP5 and the spike protein is not due to chance. The biological processes driving the production of these two proteins are likely tightly linked. Further study could provide deeper insights into how these proteins interact in vivo.

Other genes like nsp12 and nsp6 also display high mutation counts across various clades, though these are generally lower than the non-synonymous mutations. This pattern suggests that while synonymous mutations accumulate in these regions, they likely do not disrupt the gene's functional roles, reflecting the virus's overall genetic drift rather than direct adaptive changes (Ravi et al., 2024).

The Omicron variant has evolved rapidly since its initial detection in December 2021, resulting in multiple sub-lineages such as BA.2, CJ.1.1, XBB, XW, and GP.2 with varying mutations and genetic characteristics. Each sub-lineage accumulates unique mutations, often in the Spike protein. These mutations highlight the ongoing evolution and adaptation of the virus, which may lead to differences in how each sub-lineage interacts with the human immune system and spreads among populations.

The central intersection showing 48 common features across all five variants indicates shared mutations that likely confer a core set of characteristics to the Omicron variant. The Venn diagram effectively visualizes the unique and shared features among these five Omicron variants, indicating both the diversity and commonality within their genetic makeup.

The close clustering of certain variants, like BA.2 and its sub-lineages, suggests recent divergence and high genetic similarity within these groups. Recombinant variants like XBB and XW demonstrate how different Omicron sub-lineages can exchange genetic material leading to new hybrid variants.

For `98_EPI_ISL_8,144,260_BA.1.18` and `IPM20411287_EPI_ISL_12,590,756_BA.1.1` Omicron variants with high mutations in their S proteins, possible evolutionary pressures enhancing transmissibility or immune escape might be possible. These strains have nearest neighbors originating from England, specifically from late 2021, a period marked by rapid global transmission of the Omicron variant (Ren et al., 2022).

Strains IPM20407398_EPI_ISL_10,862,769_BA.1 and NIH-1247_EPI_ISL_17,667,711_BQ.1.1.15 with lower percentage in the S protein indicate different evolutionary path that conserve certain Spike features as has been stated by Oyola (2023).

The XBV lineage is positioned in the phylogenetic tree between two distinct groups. On one side, it is flanked by GP.2, BN.1.2, and CJ.1.1 lineages, while on the other side, it is closely positioned next to XBB.1 and XBB.1.5.

This arrangement suggests that XBV lineage may have evolutionary ties or shared ancestry with GP.2, BN.1.2, CJ.1.1, XBB.1 and XBB.1.5 neighboring lineages, highlighting a complex web of relationships within the Omicron variant.

The close proximity of XBV to both GP.2, BN.1.2, and the XBB lineages in the phylogenetic tree suggests that these lineages may have emerged in regions with similar evolutionary pressures. Indeed, the geographical origins reveal that several of these lineages, particularly XBV, GP.2, CJ.1.1, and XW, are primarily associated with England.

Furthermore, analysis of the overall distribution of Moroccan omicron strains reveals several strains that exhibit perfect alignment both geographically and temporally, indicating a direct evolutionary relationship within the same region and time period. Notably, strains such as ID EPI_ISL_8,144,260, ID EPI_ISL_10,862,769, and ID EPI_ISL_12,590,756 originate from England and were collected on 2021–12–19, 2021–12–25, and 2022–02–28, respectively.

Additionally, ID EPI_ISL_13,259,121 from Denmark and ID EPI_ISL_13,259,124 from Switzerland were both collected in May 2022, while ID EPI_ISL_13,421,946 and ID EPI_ISL_13,526,796 from Germany were collected in January and June 2022. This underscores the significance of regions like England, Germany, and Denmark as critical hubs for the evolution and spread of the Omicron variant. These strains reflect the close temporal and geographical connections that facilitated the variant's propagation during these key periods and underscores their key role in shaping the evolutionary trajectories of SARS-CoV-2, highlighting the importance of localized evolutionary pressures, such as population immunity and environmental conditions, in driving the observed genetic diversity within these lineages.

The XBV lineage exhibits a unique mutation profile that emphasizes changes in NSPs rather than the Spike protein or SubgARNs. This contrasts with lineages like XBB.1 and XBB.1.5, which are heavily mutated in the Spike region to enhance immune escape (Giancotti et al., 2024).

## 5 Conclusion

The comprehensive analysis of 1989 SARS-CoV-2 genome sequences from Morocco provides critical insights into the virus's evolution over a four-year period, marked by the rise of the Omicron variant and its sub-lineages. The findings reveal significant genetic diversity, with the GRA clade dominating since 2021 due to its high number of mutations, particularly non-synonymous ones in the Spike protein. These mutations are likely driven by selective pressures, including immune escape and increased transmissibility. The study also underscores the crucial role of genomic surveillance in identifying emerging variants and guiding public health responses. The correlations between viral structural and non-structural proteins suggest potential avenues for future research into the mechanisms of viral assembly and replication. Continued vigilance in monitoring SARS-CoV-2 evolution is essential to mitigate the impact of future variants.

## CRediT authorship contribution statement

**Hamza GHAMMAZ:** Writing – original draft, Visualization, Software, Methodology. **Marouane MELLOUL:** Supervision, Methodology. **Ahlam MBARKI:** Visualization, Software, Data curation. **Mouhssine HEMLALI:** Investigation, Formal analysis, Data curation. **Taha CHOUATI:** Software, Resources. **Hicham EL ANNAZ:** Investigation. **Nadia TOUIL:** Supervision, Project administration. **Mostafa ELOUENNASS:** Resources. **Khalid ENNIBI:** Methodology, Investigation. **Elmostafa EL FAHIME:** Validation, Supervision, Project administration, Conceptualization.

## Declaration of competing interest

The authors declare that they have no known competing financial interests or personal relationships that could have appeared to influence the work reported in this paper.

## Data Availability

Data will be made available on request.

## Appendix Supplementary materials

Image, application 1
