# Supplementary material for: Genomic evolution of SARS-CoV-2 in Morocco: Insights from whole genome sequences collected from 2020 to 2024
Source: Virus Res. 2025 Jan 31;353:199530. doi: 10.1016/j.virusres.2025.199530 (PMC11841124; doi:10.1016/j.virusres.2025.199530)
Supplement: Supplementary file 1 [file mmc1.docx]

Supplementary material 1 (S1): Mutations list of Moroccan SARS-CoV-2 strains

| **Gene** | **Mutation** | **Aa change** | **Count** |
| --- | --- | --- | --- |
| **NSP1** | E37D(5), D147Y(3), G98del(3), V86del(3), Y97del(3), G82del(2), H81del(2), H83del(2), M85del(2), Q22E(2), Q96del(2), T12I(2), V84del(2), E148D(1), E87D(1), F143del(1), F143L(1), G49C(1), G7V(1), H110Y(1), H165Y(1), I95del(1), K120N(1), K141del(1), K141R(1), L18F(1), L21*(1), P62S(1), P6L(1), R124C(1), R24C(1), S142del(1), V106I(1), V54I(1). | 34 | 54 |
| **NSP2** | T85I(27), K333R(25), G262S(24), P129L(10), I514T(8), K81N(7), K111R(6), L550F(6), A205V(5), A318V(5), I251M(5), L501F(5), Q588L(5), G339S(4), G481S(4), G548C(4), L24F(4), A385V(3), T44I(3), Y16H(3), A28D(2), A488V(2), E66G(2), G265V(2), H194Y(2), H237R(2), K112N(2), L274F(2), L553Q(2), N280Y(2), P129S(2), P191L(2), T634I(2), A127V(1), A159T(1), A247V(1), A336V(1), A360V(1), A510V(1), C475Y(1), D114G(1), D23Y(1), D391G(1), E162K(1), E292D(1), E453D(1), E457D(1), E460D(1), E467G(1), E57G(1), E592K(1), F10L(1), F116L(1), F226I(1), F300L(1), F455C(1), F455L(1), G212C(1), G220S(1), G458*(1), G88E(1), H208Y(1), H237Y(1), I293V(1), I296N(1), I296V(1), I387T(1), I472V(1), K171N(1), K384N(1), K454Q(1), K500N(1), L213F(1), L314S(1), L462P(1), M418T(1), M418V(1), M551I(1), P13H(1), P13S(1), Q134L(1), Q427H(1), R362L(1), R380H(1), R52C(1), S203N(1), S211F(1), S299R(1), S32L(1), S358L(1), S36F(1), S591K(1), S591R(1), T139N(1), T160I(1), T215I(1), T223I(1), T434I(1), T44P(1), T632I(1), V157F(1), V284I(1), V364I(1), V425F(1), W60*(1). | 105 | 261 |
| **NSP3** | A488S(183), P1469S(168), P1228L(154), K38R(136), A1892T(129), S1265del(126), T183I(115), A890D(114), I1412T(110), P822L(26), P74Q(25), I310T(14), M560I(12), V1229F(10), H1274Y(9), T1830I(9), S1285F(7), E426A(6), Q203H(6), S1406F(6), V473F(6), A991T(5), D218E(5), D270A(5), D782N(5), M102I(5), S1817L(5), E545G(4), I733V(4), K1260E(4), K429N(4), P778S(4), T1189I(4), T1306I(4), V325F(4), A1305V(3), A264V(3), A655V(3), E1294D(3), G1423S(3), K1199R(3), L1035I(3), M1562I(3), P153L(3), Q180H(3), S1682F(3), T1456I(3), T749I(3), V1113L(3), A1321V(2), A1711V(2), A1766V(2), A1819V(2), A564V(2), D91G(2), E1270K(2), F1391L(2), F1823L(2), G1217R(2), G283S(2), H295Y(2), K1660E(2), K1693N(2), K1771R(2), L1685F(2), M951I(2), N303S(2), P1103S(2), P662L(2), R646W(2), S1843F(2), S209G(2), S403L(2), T1004I(2), T1446I(2), T182I(2), T237A(2), T350I(2), T424I(2), T720I(2), T724I(2), T936I(2), V1315I(2), V1795F(2), V477F(2), V628I(2), A1033D(1), A1105S(1), A1105V(1), A1120S(1), A1165V(1), A1311V(1), A149V(1), A1557G(1), A1769V(1), A1818V(1), A1883V(1), A1941T(1), A225V(1), A256V(1), A338T(1), A416V(1), A480V(1), A534V(1), A644T(1), A644V(1), A991V(1), D1075G(1), D1095G(1), D110H(1), D1121Y(1), D1153G(1), D1208G(1), D1242G(1), D1318G(1), D136N(1), D178Y(1), D505A(1), D806N(1), D821N(1), D853Y(1), E1232D(1), E1270D(1), E1487K(1), E154G(1), E1799K(1), E1801G(1), E195D(1), E318K(1), E522D(1), E681A(1), F1207L(1), F1687L(1), F320L(1), F709L(1), G116C(1), G1255V(1), G1433D(1), G1440A(1), G1447V(1), G212C(1), G277E(1), G633D(1), G713C(1), G730S(1), H342Y(1), H682R(1), H682Y(1), H792Y(1), I1045N(1), I1514V(1), I1551T(1), I1683T(1), I335V(1), I341T(1), I483V(1), I591F(1), I62V(1), I789K(1), K1060R(1), K1211N(1), K1330R(1), K1382N(1), K1715R(1), K1909N(1), K384N(1), K711R(1), K837N(1), L1244F(1), L1361I(1), L1418I(1), L216F(1), L431H(1), L65M(1), L689F(1), L862F(1), L863I(1), M1218T(1), M1441I(1), M1547I(1), M1788I(1), M494I(1), M770T(1), M84I(1), N1247S(1), N1329D(1), N1438I(1), N1610I(1), N22K(1), N321I(1), P109L(1), P1159L(1), P1237S(1), P1261S(1), P1292L(1), P1292S(1), P1442S(1), P153S(1), P1719S(1), P1787S(1), P236L(1), P236S(1), P389L(1), P389S(1), P679H(1), P679L(1), P874S(1), Q1216L(1), Q1530H(1), Q167R(1), Q322H(1), Q704*(1), Q974H(1), Q995R(1), R568C(1), R586P(1), R646Q(1), R748S(1), S1087F(1), S1212L(1), S126L(1), S1375F(1), S1455F(1), S1644G(1), S1717L(1), S1913R(1), S454N(1), S650F(1), S702F(1), S716N(1), T1022I(1), T1036I(1), T1046A(1), T1056I(1), T1063I(1), T1251I(1), T1269I(1), T1348I(1), T1365I(1), T1378P(1), T1482I(1), T237I(1), T353I(1), T492I(1), T504I(1), T526I(1), T749A(1), T771I(1), T787I(1), T860L(1), T864I(1), T943I(1), T955I(1), T970M(1), V1298I(1), V1346I(1), V1420F(1), V1612F(1), V1722C(1), V1724G(1), V1762A(1), V1828A(1), V267F(1), V267I(1), V381A(1), V453I(1), V477A(1), V747L(1), V765A(1), Y1157H(1), Y129N(1), Y1323del(1), Y1463H(1). | 273 | 1736 |
| **NSP4** | T492I(307), V167L(176), V16L(25), A446V(23), V20I(12), C296F(4), F17L(4), F121L(3), D217N(2), G224S(2), L264F(2), M324I(2), P371S(2), V293L(2), V334A(2), A128V(1), A231V(1), A307V(1), A446T(1), A466T(1), A474T(1), A58V(1), A69T(1), C296G(1), E310K(1), F201L(1), F409C(1), G130V(1), G287D(1), I23N(1), I289V(1), I363M(1), I383L(1), I49V(1), K12T(1), L323F(1), L349F(1), L486V(1), M33I(1), P136S(1), R249I(1), R306K(1), S137L(1), S163A(1), S410N(1), S481L(1), S59P(1), T109M(1), T14I(1), T173I(1), T204I(1), T269I(1), T327S(1), T60I(1), T83I(1), V180I(1), V210I(1), V212L(1), V293I(1), V294K(1). | 60 | 613 |
| **NSP5** | P132H(139), K90R(16), Q83K(3), A260V(2), A129G(1), A193V(1), A234S(1), G179D(1), G71S(1), I213V(1), L75F(1), M162I(1), M49I(1), N142S(1), N221T(1), N228S(1), P108L(1), R105H(1), R222Q(1), R298C(1), S81F(1), V125I(1), V157L(1), V296D(1), V68I(1). | 25 | 181 |
| **NSP6** | G107del(243), S106del(243), T77A(163), I189V(143), L105del(125), F108del(118), V149A(24), L37F(16), T181I(14), L260F(8), A54S(4), V149F(3), A46V(2), M126I(2), Q160R(2), A117V(1), A128V(1), A161T(1), A166T(1), A2V(1), A56V(1), C113S(1), D112N(1), E195R(1), F45V(1), G9D(1), H11N(1), I266T(1), K109S(1), K63R(1), L125P(1), L142F(1), L142V(1), L15I(1), L16I(1), L185F(1), L22F(1), L239F(1), M143L(1), M192I(1), M254I(1), M83K(1), M83T(1), M92V(1), N156S(1), N205S(1), N82K(1), Q257H(1), R233C(1), R233P(1), S118L(1), T141M(1), V178I(1), V246I(1), Y253N(1), Y85C(1), Y85N(1). | 57 | 1152 |
| **NSP7** | A80V(1), G64A(1), G64D(1), M75I(1), T81I(1). | 5 | 5 |
| **NSP8** | I156T(3), L122I(3), P178S(3), R51C(2), T141M(2), D143G(1), E171D(1), E5D(1), I107V(1), I172G(1), I172V(1), K37N(1), K39R(1), L9F(1), Q24R(1), T145I(1), V130I(1), V34F(1). | 18 | 26 |
| **NSP9** | M101I(1), P57S(1), T109I(1), T18I(1), T24N(1), T35I(1), V7I(1), V85M(1). | 8 | 8 |
| **NSP10** | T102I(5), R134S(2), T39N(2), A26G(1), A32V(1), D106*(1), D82G(1), P84S(1), S15P(1), T111I(1), T12N(1), V108A(1), V108M(1), V21A(1), Y27H(1). | 15 | 21 |
| **NSP11** | F11V(1), S6L(1). | 2 | 2 |
| **NSP12** | P323L(681), G671S(197), P227L(43), L838I(6), G228S(4), G712V(4), P323F(4), A185S(3), V776L(3), Y521C(3), E922D(2), K91R(2), L805H(2), M196I(2), P830S(2), P94L(2), Q822H(2), S229G(2), S647I(2), T769I(2), T908I(2), A311S(1), A379P(1), A379V(1), A449V(1), A529V(1), A771V(1), A923S(1), C310Y(1), C765F(1), D153Y(1), D358E(1), D62A(1), D67V(1), D865N(1), E436G(1), E744E*(1), E811G(1), F812Y(1), G774C(1), G823D(1), H439Y(1), H816Y(1), H99Q(1), H99R(1), I171M(1), I244T(1), I536T(1), K332E(1), K369R(1), K718N(1), K91E(1), L247F(1), L302S(1), M380I(1), M629T(1), N158Y(1), N297K(1), N705D(1), P264T(1), P339S(1), P378L(1), Q57L(1), R181C(1), S520N(1), S649P(1), S6L(1), S784L(1), S913L(1), T252I(1), T26I(1), T276M(1), T644M(1), V11G(1), V182L(1), V234A(1), V587L(1), V637I(1), V667I(1), V720I(1), Y122H(1), Y867D(1). | 82 | 1031 |
| **NSP13** | P77L(206), H290Y(17), L428F(16), V193L(14), K460R(9), S589I(7), A296S(6), E261D(5), T351I(5), V247F(5), E319D(4), M233I(4), P504S(4), E365D(3), A505V(2), A598S(2), A598V(2), D583A(2), E168G(2), G206C(2), I79L(2), K218R(2), M274I(2), M576I(2), P300L(2), P419S(2), P53S(2), S259L(2), T367I(2), Y299N(2), A152S(1), A208V(1), A296T(1), D542N(1), E341D(1), E365G(1), G415A(1), G478D(1), G87D(1), H164Y(1), I151V(1), I575M(1), K414N(1), L43F(1), L581F(1), M429I(1), N86K(1), P172S(1), P234S(1), P47S(1), Q518H(1), R155L(1), R337P(1), R442Q(1), S236G(1), T127I(1), T141N(1), T153I(1), T413I(1), T588I(1), V154A(1), V241L(1), Y253H(1), Y324N(1), Y93*(1). | 65 | 372 |
| **NSP14** | A394V(180), I42V(129), P451S(41), V14L(25), L157F(22), M72I(8), P203L(8), P297S(7), P46L(7), L177F(4), T472M(4), H283R(3), R163C(3), A119S(2), I294V(2), S137I(2), V263F(2), Y260C(2), A1S(1), A504S(1), A79D(1), A85S(1), D234G(1), D324A(1), D345Y(1), D432A(1), D48N(1), E204D(1), E347G(1), F233L(1), F326L(1), F326S(1), F444L(1), H26Y(1), H486Y(1), I164T(1), K349N(1), L366F(1), L468Q(1), M153I(1), M169I(1), M195T(1), M501I(1), M57I(1), M62I(1), P121S(1), P203S(1), P24L(1), P327S(1), P70L(1), R400K(1), S374A(1), S461P(1), S461T(1), T516I(1), T75I(1), V182L(1), V405L(1), W247L(1), Y420*(1), Y420C(1). | 61 | 494 |
| **NSP15** | L331F(4), H234Y(3), I252T(3), K12N(3), V35F(3), G37V(2), L162F(2), L216S(2), S241R(2), T325A(2), V303F(2), A217S(1), D16Y(1), D282G(1), D335N(1), D39Y(1), D91N(1), E202K(1), E260D(1), G166del(1), I115L(1), I211V(1), I280T(1), I79V(1), K259R(1), L151del(1), L57F(1), M330L(1), N163D(1), P153del(1), P65S(1), Q130H(1), Q152del(1), Q196R(1), R138L(1), R257C(1), S154del(1), S261A(1), S287L(1), S328L(1), T114M(1), T120A(1), T33I(1), V127F(1), V155del(1), V172L(1), V22I(1), V291L(1), V313I(1), V66L(1), Y342N(1). | 51 | 68 |
| **NSP16** | A34V(15), V118L(11), T35I(8), K160R(7), D179G(6), S33I(6), T140I(6), E264D(3), Q238H(3), V288F(3), C154F(2), E284D(2), F193P(2), N297K(2), A116V(1), A188S(1), A188T(1), A192T(1), E173G(1), G113C(1), H186D(1), I128V(1), I169L(1), I237T(1), K170*(1), K182N(1), L262*(1), N286S(1), N298K(1), P236L(1), P236S(1), Q266R(1), R216S(1), S2G(1), T151I(1), Y242H(1). | 36 | 98 |
| **Spike** | D614G(697), T478K(313), P681H(259), H69del(253), V70del(253), Y144del(241), L452R(225), P681R(204), T19R(192), D950N(188), F157del(160), R158del(160), N501Y(155), H655Y(143), N856K(143), N679K(142), N969K(142), Q954H(142), T547K(142), D796Y(140), E156G(140), N764K(140), A67V(139), L981F(131), G339D(125), G142del(123), V143del(123), D1118H(115), T716I(115), A570D(114), S982A(114), T95I(110), S477N(100), G446S(97), K417N(97), E484A(96), Q493R(96), S373P(95), S375F(95), N440K(94), S371L(87), Q613H(84), T29A(83), T250I(79), N211del(72), T299I(70), G142D(50), Q498R(40), G496S(39), Y505H(36), A222V(29), V6F(18), D427Y(14), D843N(12), V70F(12), Q677H(11), L5F(10), S254F(10), F1109L(9), F157L(9), L585F(9), R346K(9), E484K(8), A653V(7), G769V(7), Q52R(7), L582F(6), S939F(6), V1133F(6), A243S(5), F490S(5), R78M(5), W258L(5), A1020S(4), D215G(4), F797C(4), F888L(4), G1219V(4), M1237I(4), V622F(4), A701V(3), F565L(3), I100T(3), I850L(3), L18F(3), L699I(3), Q1113K(3), V1264L(3), A262S(2), A27S(2), A520S(2), A623S(2), A852S(2), D253A(2), D88H(2), G446V(2), H49Y(2), H69Y(2), L1265F(2), L54F(2), P1263L(2), P499R(2), S12F(2), S375P(2), S494P(2), S98F(2), T1006S(2), T22I(2), T791I(2), V1176F(2), A1016T(1), A1078T(1), A1078V(1), A1174V(1), A243del(1), A344S(1), A522S(1), A626V(1), A67S(1), A688V(1), A845S(1), A845V(1), A892S(1), C1032G(1), C1250Y(1), C336R(1), D138H(1), D138Y(1), D178N(1), D80A(1), D80G(1), D839N(1), E1072G(1), E154K(1), E471D(1), E484Q(1), E554Q(1), E583D(1), E780Q(1), F1075Y(1), F157S(1), F2V(1), F329L(1), F32L(1), F347S(1), F400L(1), F464L(1), F4L(1), F565V(1), F797L(1), F79L(1), F86S(1), G1124V(1), G1251R(1), G1267V(1), G142V(1), G181A(1), G261D(1), G261V(1), G311E(1), G431R(1), G482V(1), G496G(1), G669D(1), G89D(1), G932S(1), H1058Q(1), H1083Y(1), H1271D(1), H146Q(1), I1130V(1), I1232V(1), I203M(1), I770V(1), I818V(1), I896T(1), I923M(1), I934F(1), K1191Q(1), K1255I(1), K1269R(1), K558N(1), K811D(1), K814N(1), L1063F(1), L141F(1), L18K(1), L241del(1), L242del(1), L821I(1), L922W(1), L938F(1), M1229I(1), M1229T(1), N1108S(1), N148T(1), N17K(1), N188D(1), N282K(1), N354K(1), N439K(1), N450S(1), N501I(1), N641I(1), N703S(1), N717S(1), P25T(1), P26L(1), P384L(1), P631del(1), P812R(1), P82L(1), P85Q(1), Q1071H(1), Q677R(1), R158G(1), R346G(1), R346I(1), R34P(1), S1037T(1), S1242G(1), S13I(1), S256L(1), S256P(1), S366A(1), S371F(1), S46L(1), S637Y(1), S640F(1), S659P(1), S704*(1), S813R(1), S929T(1), T1076I(1), T1273S(1), T144ins(1), T284A(1), T33I(1), T430S(1), T478I(1), T547I(1), T630del(1), T632del(1), T632S(1), T678I(1), T723S(1), T724I(1), T747I(1), T791K(1), T859I(1), V1104L(1), V1122M(1), V143 del(1), V159A(1), V16F(1), V227L(1), V341F(1), V367L(1), V3G(1), V6A(1), V826L(1), V90L(1), Y1272F(1), Y144S(1), Y145del(1), Y145N(1), Y674F(1). | 267 | 7665 |
| **NS3** | S26L(206), Q57H(57), W131C(20), L85F(15), W131L(12), T151I(11), H182Y(9), A54S(7), E102V(7), Q57R(7), D173G(6), D238Y(5), G224V(5), V112F(5), L15F(4), V202L(4), G251V(3), H78Y(3), K16T(3), L108F(3), L147F(3), S165F(3), A98T(2), D155Y(2), G100C(2), G100V(2), G172R(2), I20M(2), L106F(2), P104S(2), Q185H(2), Q38R(2), S171L(2), T271I(2), A103S(1), A31T(1), A33S(1), A33V(1), A54V(1), A98V(1), D155G(1), D155H(1), D210N(1), E226G(1), G172C(1), G174D(1), G174V(1), G18C(1), G254R(1), G44R(1), G49D(1), G49V(1), H227Y(1), I179M(1), I232V(1), I43del(1), K67R(1), K75N(1), L111I(1), L53I(1), L83F(1), L95F(1), M125I(1), M260K(1), N119H(1), P240L(1), P25S(1), P262S(1), P267S(1), R126G(1), R126S(1), R134L(1), S117G(1), S117N(1), S177T(1), S40L(1), S40P(1), S58N(1), S60F(1), S74N(1), S92L(1), T12I(1), T208A(1), T24I(1), T269M(1), T32A(1), T34M(1), T89I(1), T9K(1), V121A(1), V13L(1), V255I(1), V50I(1), V55F(1), V90F(1), V90L(1), W128C(1), W69C(1), Y145H(1). | 99 | 487 |
| **E** | T9I(141), L21F(4), V49L(4), P71L(2), S68F(2), *76Q(1), A36V(1), C40F(1), C43R(1), C44Y(1), L19P(1), L73F(1), N45R(1), P71S(1), R61H(1), S43ins(1), V47G(1), V62F(1). | 18 | 167 |
| **M** | I82T(212), A63T(132), Q19E(132), D3G(126), H148Q(25), H125Y(2), R72K(2), T208I(2), A104S(1), A2V(1), A38C(1), A63V(1), A85V(1), F103L(1), H148R(1), I32F(1), I82S(1), K15N(1), L124F(1), L13F(1), L34F(1), M109I(1), N207H(1), T175M(1), V70A(1). | 25 | 650 |
| **NS6** | E54*(4), F2del(4), D53Y(1), D61L(1), E13D(1), H3Y(1), I36V(1), P57L(1), T21I(1). | 9 | 15 |
| **NS7a** | T120I(111), V82A(109), L116F(9), E41*(7), A8V(6), Q62*(6), P34L(4), D51A(3), K53R(3), P45L(3), C113F(2), K85*(2), L12F(2), V93F(2), A105S(1), A50V(1), A55E(1), A66T(1), C58*(1), E91*(1), F101del(1), F63del(1), F6Y(1), G70A(1), H73Y(1), I100del(1), I3S(1), I3T(1), K72N(1), L56P(1), L5F(1), L77V(1), L96del(1), L96F(1), P68S(1), P84L(1), P99del(1), P99S(1), Q21R(1), Q62H(1), Q76L(1), R80I(1), R80K(1), S36P(1), S98del(1), S98F(1), T28I(1), Y97del(1). | 48 | 303 |
| **NS7b** | T40I(200), L6del(3), S5del(3), E3del(2), I2del(2), L4del(2), M24V(2), *44K(1), *44Q(1), C41F(1), C41S(1), D8del(1), H42R(1), I7del(1), M24I(1). | 15 | 222 |
| **NS8** | D119del(194), F120del(194), Q27*(118), R52I(117), Y73C(115), K68*(76), V62L(16), F120L(6), I121L(6), F120S(5), R101H(4), A65S(3), E106*(3), E19*(3), S67F(3), A65V(2), L95F(2), P30S(2), P38S(2), T11K(2), V32L(2), V62M(2), W45C(2), Y31H(2), A14T(1), A15L(1), A51V(1), D119N(1), D119Y(1), F16S(1), F41V(1), G50*(1), G8R(1), H40N(1), I121V(1), I58F(1), I71T(1), I76T(1), L109S(1), L118V(1), L4P(1), L60F(1), P36S(1), P85G(1), P85L(1), P93L(1), Q27K(1), Q29*(1), S54L(1), T87A(1), T87G(1), Y73N(1). | 52 | 909 |
| **N** | R203K(306), G204R(305), R203M(201), D63G(197), D377Y(195), G215C(170), P13L(146), E31del(128), R32del(128), S33del(128), D3L(114), S235F(114), M210I(26), A211V(25), A220V(11), T205I(11), S186Y(10), A155S(8), P46S(7), D144H(6), K373N(6), N8T(6), P207L(6), P365S(5), Q289H(5), Q9L(5), S183T(5), S2Y(5), A12G(4), D3del(4), G238C(4), P364L(4), R209I(4), R385K(4), A376T(3), D348H(3), L230F(3), M234I(3), Q9H(3), S187L(3), S190I(3), S193N(3), A12V(2), A152S(2), A156S(2), A35V(2), A414S(2), D128Y(2), G164V(2), L219F(2), N140K(2), P151S(2), P199L(2), P383L(2), Q160K(2), Q28H(2), R40S(2), R89K(2), S194L(2), S206P(2), S327L(2), T379I(2), A134V(1), A208V(1), A211S(1), A218S(1), A311S(1), A398S(1), A398V(1), A419S(1), D371H(1), D399H(1), D3Y(1), G116E(1), G179S(1), G179V(1), G204L(1), G243R(1), G25C(1), G34E(1), H59P(1), I94T(1), K169N(1), K256N(1), K373*(1), K374T(1), K375N(1), K61R(1), L139F(1), L400F(1), N27S(1), N8S(1), P142S(1), P151L(1), P168S(1), P199S(1), P20L(1), P279Q(1), P383S(1), P6H(1), P6L(1), P6T(1), Q303E(1), Q384R(1), Q409R(1), Q418P(1), R14C(1), R185C(1), R195I(1), R209K(1), R209T(1), R32L(1), R40C(1), R92K(1), S180R(1), S193I(1), S193T(1), S21P(1), S318P(1), T141P(1), T296I(1), T325I(1), T334I(1), T362I(1), T362K(1), V270L(1), V72A(1). | 127 | 2432 |
| **NS10** | V30L(11), D31Y(2), A28V(1), M1I(1), P10L(1), V32L(1), V6I(1). | 7 | 19 |
| TOTAL | | 1564 | 18991 |


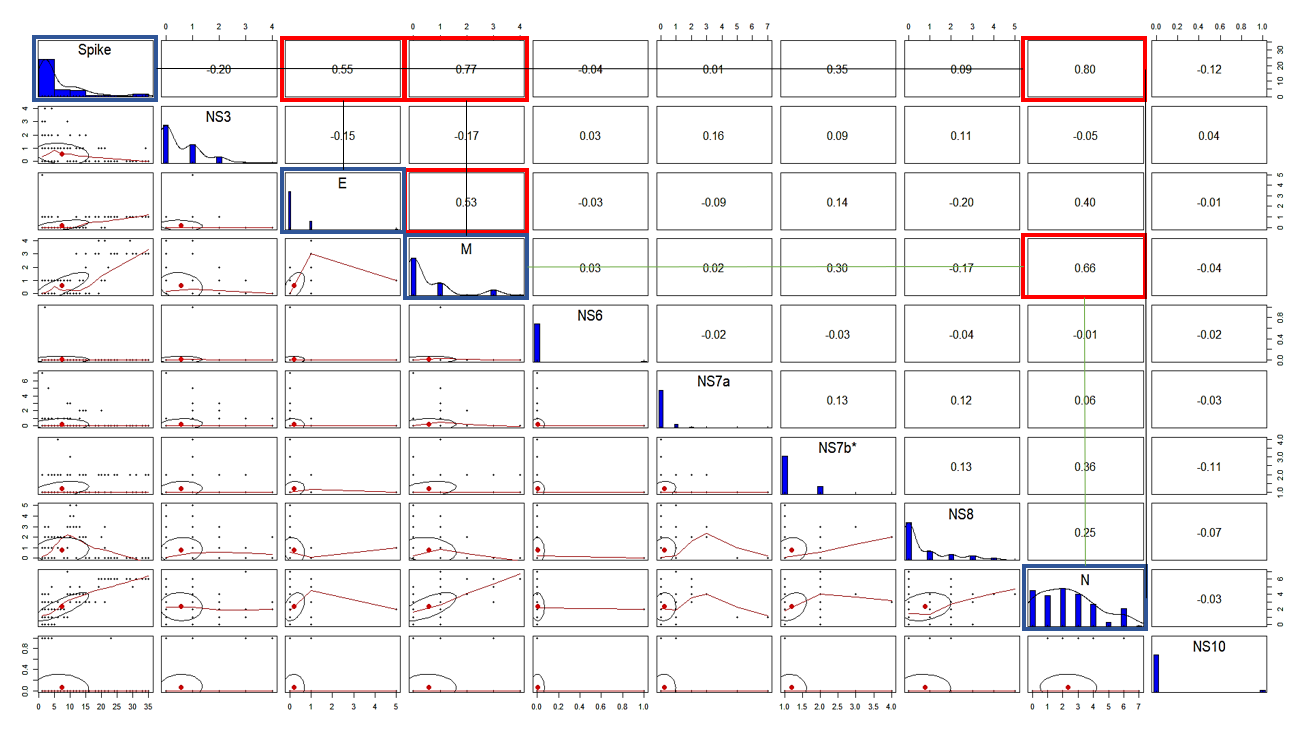

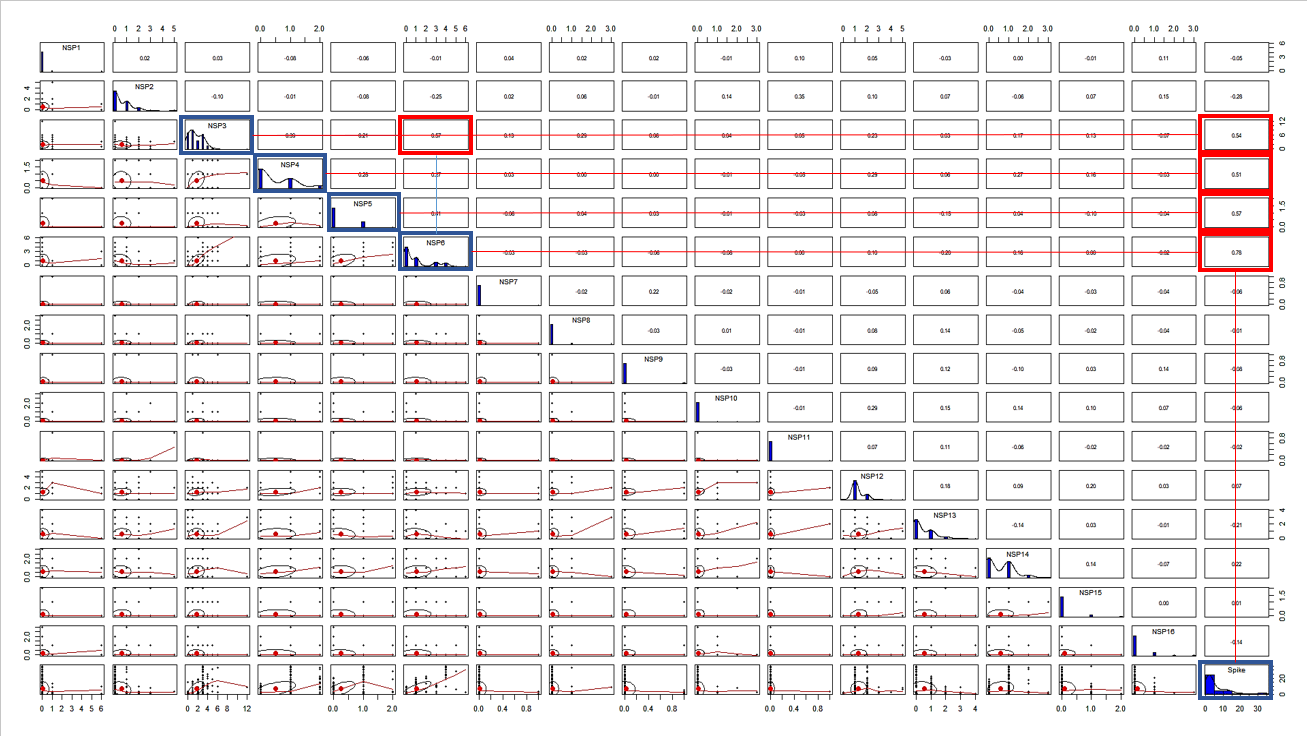


Supplementary material 2 (S2): Pearson correlation test describing the relation between (A) non-structural protein (B) structural protein and the Spike gene. The histograms on the diagonal show the distribution of each variable separately (NSP1 to NS10). Data relative to each variable does not follow a normal or Gaussian distribution. Some variables have a high correlation coefficient with the spike gene, which indicates a strong linear relationship between these variables and the spike gene. Specifically, NSP6, M, and N have correlation coefficients of 0.76, 0.77, and 0.80, respectively. On the other hand, some variables have a low correlation coefficient with the spike gene, which suggests a weak or no linear relationship. NSP13, NSP16, and NS10 have correlation coefficients of -0.21, -0.14, and -0.12, respectively.

B

A
